# Supplementary material for: Using MaxEnt to Predict the Potential Distribution of the Little Fire Ant (Wasmannia auropunctata) in China
Source: Insects. 2022 Nov 1;13(11):1008. doi: 10.3390/insects13111008 (PMC9698453; doi:10.3390/insects13111008)
Supplement: Supplementary file 1 [file insects-13-01008-s001.zip › Supplementary.pdf]

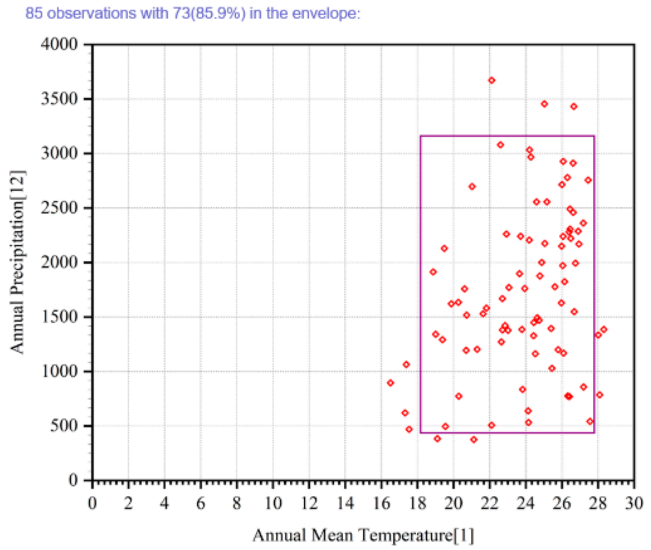

Figure S1 Environmental envelope model of recorded points of *W. auropunctata*, the envelope showing the wide range of Annual precipitation (Bio 12) against an effective small range of Annual mean temperature (Bio 1).

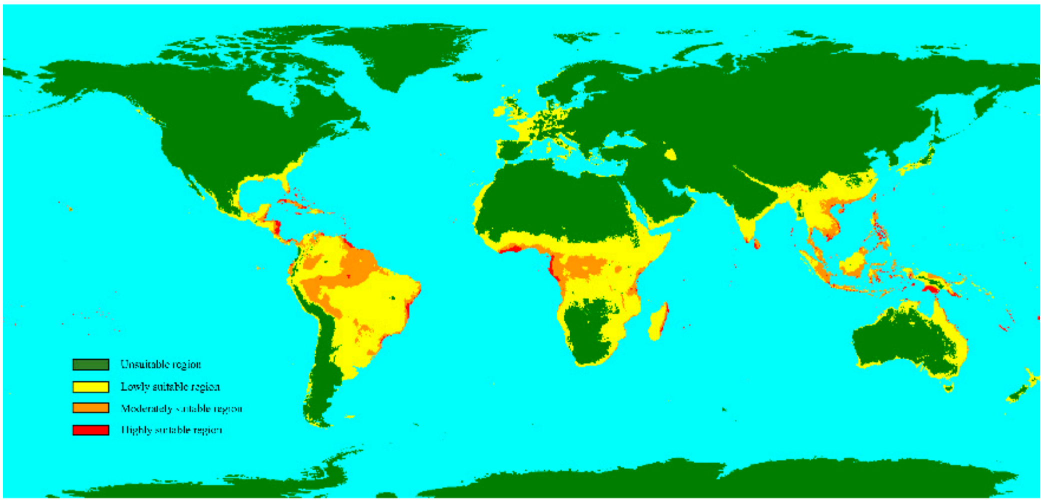

Figure S2 Potential distribution map of *W. auropunctata* in the world under the current climate environment
